# Body Weight and Breast Cancer Treatment Experiences: Results From the Share Thoughts on a Breast Cancer Study

**DOI:** 10.1002/cam4.70628

**Published:** 2025-02-04

**Authors:** Sarah H. Nash, Elizabeth Verhage, Bradley D. McDowell, Joan Neuner, Elizabeth Chrischilles, Ingrid M. Lizarraga, Mary Schroeder

**Affiliations:** ^1^ Department of Epidemiology, College of Public Health University of Iowa Iowa City Iowa USA; ^2^ Holden Comprehensive Cancer Center University of Iowa Iowa City Iowa USA; ^3^ Department of Internal Medicine Medical College of Wisconsin Milwaukee Wisconsin USA; ^4^ Department of Surgical Oncology, College of Medicine University of Iowa Iowa City Iowa USA; ^5^ Division of Health Services Research, College of Pharmacy University of Iowa Iowa City Iowa USA

**Keywords:** body mass index, cancer treatment, obesity, weight stigma

## Abstract

**Purpose:**

Differences in breast cancer recurrence and survival occur by body size; the role of treatment differences in these disparities has been underexplored. Our objective was to evaluate differences in treatments received, patient experiences of care, and treatment decision‐making processes among breast cancer survivors by body size.

**Methods:**

We used data from the Share Thoughts on Breast Cancer study. Participants (*n* = 1198) completed a survey that included information on demographics, treatments received, quality of care, and decision‐making. We used descriptive statistics to evaluate differences in survey response by BMI category, and multivariable‐adjusted multinomial and logistic regression to examine associations of BMI with treatments received.

**Results:**

Those with higher BMI were more likely to be older, report fair/poor health, not have a college‐level education, be non‐white, not be insured, have an income under $50,000, be unemployed, and report a history of several chronic diseases. Although there were unadjusted associations, after adjustment, women with obesity were not significantly less likely to receive mastectomy [OR 0.79 (0.50, 1.26) and OR 0.66 (0.38, 1.16), for BMI 30–35 and 35+ kg/m^2^ respectively] or contralateral prophylactic mastectomy [OR 0.92 (0.59, 1.44) and OR 0.80 (0.46, 1.39)] than those without obesity. Similarly, we found no association of BMI with reconstructive surgery [OR 0.97 (0.58, 1.60) and OR 0.58 (0.30, 1.11)] after adjustment. Women with obesity were less likely to report that their breast cancer care was excellent or very good (*p* = 0.026).

**Conclusions:**

We observed no differences in breast cancer treatments received by BMI after adjustment for key covariates in this study sample. Further research is necessary to determine why quality of care may be perceived as lower among women with obesity.

**Abbreviations**
BMI: body mass index
ER: estrogen receptor
GPC: Greater Plains Collaborative
IRB: Institutional Review Board

## 1 Introduction

Women with obesity may be at higher risk of developing post‐menopausal breast cancer and breast cancer recurrence [1, 2], as well as experience lower disease free and overall cancer survival [3]. This increased risk has been attributed, at least in part, to the effect of obesity on estrogen metabolism, since associations are strongest with estrogen receptor (ER) positive and post‐menopausal cancers [4]. Further, obesity is a risk factor for other comorbidities that impact patients' ability to receive and complete all modalities of treatment [5, 6], which in turn impacts recurrence risk [7].

However, differences in breast cancer diagnoses and outcomes [8, 9] by weight may also be linked with access to and utilization of healthcare services including cancer treatment; this phenomenon is not well understood. Evidence does suggest that women with obesity are less likely to receive appropriate breast cancer screening services [10], which may result in later‐stage diagnoses. Furthermore, women with obesity may be less likely to receive full chemotherapy doses [11, 12, 13], and/or may be more likely to experience surgical complications [14, 15]. In addition to consideration of co‐morbidities and medical complications associated with excess skin and adipose tissue, patients with obesity also experience stigma and weight‐based implicit and explicit bias by those in the medical profession that may impact decision‐making and treatments received [16]. In order to address weight‐based disparities in breast cancer outcomes, it is necessary to understand whether patients with obesity receive different treatments than those without obesity, as well as whether they experience the decision‐making and treatment process differently.

In this study, our primary objective was to examine whether patient‐reported breast cancer treatment varied across body mass index (BMI) in a multi‐site sample of patients diagnosed and treated primarily in the Midwest. Our secondary objective was to describe patient experiences of care and decision‐making by BMI. We used data from the Greater Plains Collaborative (GPC) “Share Thoughts on Breast Cancer” study, which examined patient‐reported breast cancer care experiences and quality of life among survivors an average of 2 years after diagnosis.

## 2 Methods

### 2.1 Ethics Statement

The University of Iowa Institutional Review Board (IRB) approved the Share Thoughts on Breast Cancer study protocol. The IRBs for the following collaborating medical centers deferred to the Iowa IRB under the GPC reliance agreement: University of Texas Southwestern Medical Center; University of Kansas Medical Center; University of Wisconsin Carbone Cancer Center; University of Nebraska Medical Center; University of Minnesota; Medical College of Wisconsin; and Marshfield Clinic Research Foundation.

### 2.2 Data Source and Study Population

Recruitment and study procedures for the Share Thoughts on Breast Cancer study have been described elsewhere [17]. Briefly, using tumor registry data from their respective institutions, each medical center identified all patients aged 18+ who had been diagnosed with breast cancer during the study period of January 2013 to May 2014. Eligible individuals were women with microscopically confirmed ductal carcinoma in situ and stage 0–III breast cancer diagnosed during the study period. Women who had previously been diagnosed with cancer or had passed away between the time of diagnosis and survey were excluded.

A total of 250 patients from each of the seven centers were randomly selected for the survey sample; two sites had fewer than 250 eligible patients, so all eligible patients from those sites were selected for the survey sample. Each center mailed all study materials in a single packet containing a cover letter, a 21‐page questionnaire, medical record consent, and a $10 incentive over a 6‐week period beginning June 19, 2015. The Share Thoughts on Breast Cancer survey instrument has been previously described [17], and it included validated questions spanning a wide range of topics. The sections used in this study included those on self‐reported general health, goals of therapy, treatments received, interactions with cancer care providers, and perceived quality of care. One remailing to non‐respondents was conducted 4 weeks after the initial mailing. A total of 1986 patients were invited to participate, and 1235 (62.2%) responded to the survey. Because we were specifically interested in differences related to excess body weight, we excluded patients who reported having a BMI of less than 18.5 kg/m^2^ at the time of their breast cancer diagnosis. Our final sample size was 1198 participants.

### 2.3 Variable Definitions

Our primary independent variable was obesity, operationalized using BMI at time of diagnosis. BMI was calculated based on self‐reported height and weight that participants reported for the time of their breast cancer diagnosis (*not* time of survey) using the formula BMI = weight (kg)/height (m)^2^. BMI categories were defined as 19–24, 25–29, 30–35, 36+ kg/m^2^ [18].

We examined variations in several outcomes by BMI category grouped into: treatments received, perceived quality of treatment, and treatment goals and decision‐making. Analyses assessing differences in the types of surgical treatment received focused on most extensive treatments received and were restricted to only those individuals who received surgical treatment. Secondary analyses of differences in patient experiences of care included perceived care coordination and responsiveness, overall quality of care, and provider communication. We constructed a care coordination and responsiveness scale based on the work of Ayanian et al. [19] by summing the responses from six questions that used a Likert‐type scale as described above. Differences between actual and preferred decision‐making were calculated as previously described [20].

Patient characteristics included a range of demographic and clinical variables, including age at diagnosis, self‐rated health, smoking history, education, marital status, race, insurance, income, employment status, comorbidity history, and health literacy. We created binary variables for smoking (never vs. ever), education (college graduate vs. not), marital status (married/partner vs. not), race (white vs. nonwhite), insured (yes vs. no), and employment status (yes vs. no). Age at diagnosis was calculated using self‐reported birthdate and date of diagnosis. Health literacy was assessed using three questions: (1) “How often do you have someone help you read hospital materials”, (2) “How often can you fill out medical forms by yourself”, and (3) “How often do you have problems learning about your medical condition because of difficulty understanding written information”. Patients responded to each question on a 5‐point scale from “Always” (0) to “Never” (4) with the second question reverse coded. These values were summed to create a health literacy score that ranged from 0 to 12 with higher values indicating higher levels of health literacy [21]. The burden of comorbidity was summarized by creating a count variable ranging from 0 to 9 for the number of pre‐existing conditions reported from a list including bronchitis, heart disease, other cancer, diabetes, hypertension, stroke, arthritis, high cholesterol, and a prior rotator cuff problem; a higher score indicated a greater number of comorbid conditions. Self‐rated health was rated on a scale from poor to excellent and grouped into three categories: excellent/very good, good, and fair/poor.

### 2.4 Statistical Analyses

We generated descriptive statistics for demographic and outcome variables by the BMI category and tested for statistical significance using Cochrane–Mantel–Haenszel chi‐squared tests for trend. We used multivariable‐adjusted regression models to examine associations of BMI with treatments received. The first model (model 1) was a multinomial model with lumpectomy (reference group), mastectomy, and bilateral mastectomy as outcomes. The second model (model 2) was a logistic regression model with the outcome of reconstructive surgery received (yes/no). In a separate model, we examined associations with receipt of reconstruction only among those who received a mastectomy or bilateral mastectomy. For both models, the BMI category was the primary exposure, and age, self‐reported health, education, marital status, insurance, income, health literacy score, and site of recruitment/treatment were included in the model as covariates. Comorbidities were not included as covariates in these models due to concern for overfitting and collinearity with self‐reported health. Model 2 was additionally adjusted for initial surgery received (i.e., lumpectomy or mastectomy). We conducted unadjusted descriptive analyses examining differences in treatment quality, decision‐making, and care coordination variables using Cochrane–Mantel–Haenszel chi‐squared tests for trend. All analyses were conducted using SPSS v29.0.1.0 (IBM Corp. Released 2021. IBM SPSS Statistics for Mac, Version 28.0.1.0 Armonk, NY: IBM Corp). Statistical significance was set at *α* = 0.05.

## 3 Results

### 3.1 Demographic and Clinical Characteristics

Table 1 gives demographic and clinical characteristics of study participants by the BMI category. BMI ranged from 19 to 62 kg/m^2^. We observed several differences between groups: women with overweight or obesity were older; less likely to report being in excellent or very good health; less likely to be college graduates; more likely to be divorced, widowed, or single; less likely to have private health insurance; and less likely to report earning over $50,000 per year in income. Women with a BMI over 36 kg/m^2^ were less likely to be white. There were also differences in self‐reported health history: women with obesity were more likely to report a previous diagnosis of bronchitis, heart disease, diabetes, hypertension, arthritis, and high cholesterol.

**TABLE 1:** Demographic and clinical characteristics of Share Thoughts on Breast Cancer study participants, Greater Plains Collaborative, 2013–2014, *n* = 1198.

Covariate	Level	BMI 19–24	BMI 25–29	BMI 30–35	BMI 36+	*p* ^a^
		*N* = 417	*N* = 353	*N* = 262	*N* = 166	
		*n* (%)	*n* (%)	*n* (%)	*n* (%)	
Demographics						
Age at diagnosis (years)	< 45	75 (18.5)	46 (13.4)	19 (7.5)	12 (7.4)	**< 0.001**
	45–54	128 (31.6)	82 (23.8)	55 (21.7)	32 (19.8)	
	55–64	106 (26.2)	97 (28.2)	84 (33.1)	56 (34.6)	
	65+	96 (23.7)	119 (34.6)	96 (37.8)	62 (38.3)	
Self‐rated health	Excellent/VG	291 (70.1)	218 (61.9)	127 (48.8)	49 (30.1)	**< 0.001**
	Good	100 (24.1)	114 (32.4)	105 (40.4)	86 (52.8)	
	Fair/poor	24 (5.8)	20 (5.7)	28 (10.8)	28 (17.2)	
History of smoking	Never smoked	253 (61.0)	217 (61.5)	153 (58.4)	97 (58.4)	0.44
	History of smoking	162 (39.0)	136 (38.5)	109 (41.6)	69 (41.6)	
College graduate	College graduate or higher	213 (51.6)	185 (52.6)	89 (34.4)	58 (35.4)	**< 0.001**
	Not a college graduate	200 (48.4)	167 (47.4)	170 (65.6)	106 (64.6)	
Married/partner	Married/partner	324 (77.7)	260 (73.7)	176 (67.7)	103 (62.0)	**< 0.001**
	Divorced/widowed/single	93 (22.3)	93 (26.3)	84 (32.3)	63 (38.0)	
Race	White (vs. nonwhite)	393 (94.2)	323 (91.5)	245 (93.5)	144 (86.7)	**0.017**
Insurance	Not insured	4 (1.0)	10 (2.8)	3 (1.2)	4 (2.4)	**< 0.001**
	Medicaid	15 (3.6)	14 (4.0)	16 (6.2)	18 (10.9)	
	Medicare	90 (21.8)	109 (31.1)	85 (32.7)	57 (34.5)	
	Private insurance	303 (73.5)	218 (62.1)	156 (60.0)	86 (52.1)	
Income	Under $50,000	108 (26.2)	118 (34.3)	103 (40.2)	72 (44.7)	**< 0.001**
	Over $50,000	246 (59.7)	182 (52.9)	117 (45.7)	67 (41.6)	
	Refused	58 (14.1)	44 (12.8)	36 (14.1)	22 (13.7)	
Employed at the time of diagnosis	Yes	250 (60.4)	205 (58.4)	144 (55.2)	80 (48.5)	**0.009**
Health literacy score (0–12)	Mean (StDev)	10.5 (1.9)	10.3 (2.0)	10.1 (2.1)	10.2 (2.4)	0.062
Personal health history						
History of bronchitis	Yes	15 (3.6)	15 (4.3)	26 (10.0)	15 (9.4)	**< 0.001**
History of heart disease	Yes	13 (3.1)	9 (2.6)	9 (3.5)	13 (8.1)	**0.023**
History of cancer	Yes	15 (3.6)	10 (2.8)	12 (4.6)	7 (4.3)	0.51
History of diabetes	Yes	16 (3.9)	21 (6.0)	30 (11.6)	47 (29.0)	**< 0.001**
History of hypertension	Yes	97 (23.3)	119 (33.7)	129 (49.6)	115 (70.6)	**< 0.001**
History of stroke	Yes	6 (1.5)	6 (1.7)	5 (1.9)	4 (2.5)	0.41
History of arthritis	Yes	86 (20.8)	94 (26.6)	98 (37.8)	81 (50.3)	**< 0.001**
History of high cholesterol	Yes	97 (23.4)	131 (37.3)	116 (44.8)	77 (47.5)	**< 0.001**
Prior rotator cuff diagnosis	Yes	56 (13.4)	42 (11.9)	42 (16.2)	32 (19.8)	0.053
Comorbidity count	Mean (Std)	1.0 (1.2)	1.3 (1.3)	1.8 (1.4)	2.4 (1.7)	**< 0.001**

^a^ Parametric *p*‐values calculated using Mantel–Haenszel chi‐square test for trend for categorical variables and ANOVA for linear variables. Variable totals may differ due to missing data by variable (variables < 3% missing). Bold values indicate statistically significance.

### 3.2 Associations of BMI With Surgeries Received

We observed differences in most extensive surgery received by the BMI category (Table 2); a smaller proportion of women with higher BMIs received a bilateral mastectomy than those with lower BMIs (e.g., 27% among BMI 19–24 kg/m^2^ vs. 18% among BMI 36^+^ kg/m^2^), and a higher proportion received a lumpectomy (e.g., 54% among BMI 19–24 kg/m^2^ vs. 64% among BMI 36^+^ kg/m^2^). Women with higher BMIs were less likely to have received more extensive surgery after initial surgical treatment (e.g., 11% among BMI 19–24 kg/m^2^ vs. 8% among BMI 36^+^ kg/m^2^) and less likely to report having received reconstructive surgery (e.g., 38% among BMI 19–24 kg/m^2^ vs. 20% among BMI 36^+^ kg/m^2^). Table 3 presents univariable and multivariable‐adjusted associations of BMI and other covariates with most extensive surgery (model 1) and receipt of reconstructive surgery (model 2). While BMI was associated with reduced odds of bilateral mastectomy in univariable models [OR (95% CI): 30–35 kg/m^2^ = 0.62 (0.42, 0.92); 36^+^ kg/m^2^ = 0.54 (0.34, 0.86)], we observed no significant associations of BMI with receipt of mastectomy or bilateral mastectomy (vs. lumpectomy) after adjustment for covariates. Similarly, while higher BMI was associated with reduced odds of receipt of reconstructive surgery in unadjusted models [OR (95% CI): 30–35 kg/m^2^ = 0.56 (0.40, 0.79); 36^+^ kg/m^2^ = 0.41 (0.26, 0.63)], after adjustment for covariates, there was no association. Prior surgeries received was the strongest predictor of whether an individual received reconstruction [aOR (95% CI): mastectomy = 16.3 (10.3, 25.9); bilateral mastectomy = 29.1 (18.5, 45.7)]. Odds ratios were substantially lower than 1.0 for all BMI categories above 25 kg/m^2^ in both fully adjusted models; however, these associations failed to reach statistical significance, which may be a reflection of small cell sizes. In a model examining associations with reconstructive surgery only among those who received mastectomy or bilateral mastectomy (Table S1), we observed reduced odds of receiving reconstruction among all women with overweight or obesity in univariate models [OR (95% CI): 25–30 kg/m^2^ = 0.56 (0.36, 0.88); 30–35 kg/m^2^ = 0.48 (0.29, 0.81); 36^+^ kg/m^2^ = 0.35 (0.19, 0.65)]. Once again, odds ratios were substantially lower than 1.0 for all BMI categories above 25 kg/m^2^ in fully adjusted models; however, these associations failed to reach statistical significance. We observed no differences in receipt of chemotherapy, hormone therapy, or receipt of/ plans to have genetic testing by the BMI category (data not shown).

**TABLE 2:** Breast cancer surgeries reported among participants of the Share Thoughts on Breast Cancer Study, stratified by BMI category, 2013–2014, *n* = 1198.

Covariate	Level	BMI 19–24	BMI 25–29	BMI 30–35	BMI 36+	*p* ^b^
		*n* = 417	*n* = 353	*n* = 262	*n* = 166	
Most extensive surgery^a^	Bilateral mastectomy	114 (27.3)	86 (24.4)	51 (19.5)	29 (17.5)	**< 0.001**
	Mastectomy	72 (17.3)	73 (20.7)	42 (16.0)	27 (16.3)	
	Lumpectomy	223 (53.5)	189 (53.5)	160 (61.1)	106 (63.9)	
	No surgery	8 (1.9)	5 (1.4)	9 (3.4)	4 (2.4)	

	*n* (%Yes)	*n* = 409	*n* = 348	*n* = 253	*n* = 162	
Among those who received surgery						
Received more extensive surgery after initial surgical treatment		44 (10.8)	34 (9.8)	8 (3.2)	13 (8.0)	**0.015**
Received reconstructive surgery		154 (37.7)	112 (32.2)	64 (25.3)	32 (19.8)	**< 0.001**

^a^ Patients could report more than one surgery; here, give the most extensive of all surgeries reported by each patient.

^b^ Parametric *p*‐values calculated using the Mantel–Haenszel chi‐square test for trend. Bold values indicate statistically significance.

**TABLE 3:** Results of multivariable analyses examining associations with receipt of mastectomy/bilateral mastectomy versus lumpectomy (multinomial model; model 1) and surgical reconstruction (model 2), Share Thoughts on Breast Cancer study participants who received surgery, Greater Plains Collaborative, 2013–2014, *n* = 1172.

Model outcome →	Model 1: multinomial model (reference = lumpectomy)				Model 2: logistic regression model	
	Mastectomy unadjusted OR	Mastectomy aOR	Bilateral mastectomy unadjusted OR	Bilateral mastectomy aOR	Reconstruction unadjusted OR	Reconstruction aOR
Variable/level ↓						
BMI (reference = 19–24 kg/m^2^)						
25–29	1.2 (0.82, 1.75)	1.20 (0.80, 1.79)	0.89 (0.63, 1.25)	1.16 (0.79, 0.70)	0.79 (0.58, 1.06)	1.00 (0.65, 1.55)
30–35	0.81 (0.53, 1.25)	0.79 (0.50, 1.26)	**0.62 (0.42, 0.92)**	0.92 (0.59, 1.44)	**0.56 (0.40, 0.79)**	0.97 (0.58, 1.60)
36+	0.79 (0.48, 1.3)	0.66 (0.38, 1.16)	**0.54 (0.34, 0.86)**	0.80 (0.46, 1.39)	**0.41 (0.26, 0.63)**	0.58 (0.30, 1.11)
Age category (reference = < 45 years)						
45–54 years	0.73 (0.40, 1.34)	0.75 (0.40, 1.39)	**0.31 (0.20, 0.48)**	**0.34 (0.21, 0.53)**	**0.41 (0.27, 0.62)**	0.66 (0.38, 1.14)
55–64 years	0.67 (0.37, 1.20)	0.70 (0.38, 1.27)	**0.15 (0.09, 0.23)**	**0.17 (0.10, 0.27)**	**0.18 (0.12, 0.27)**	**0.33 (0.19, 0.57)**
65+ years	**0.53 (0.30, 0.95)**	**0.37 (0.15, 0.89)**	**0.06 (0.03, 0.09)**	**0.03 (0.01, 0.09)**	**0.03 (0.02, 0.06)**	**0.14 (0.05, 0.37)**
Self‐reported health (reference = excellent/very good)						
Good	1.31 (0.94, 1.80)	1.32 (0.92, 1.90)	0.65 (0.47, 0.89)	0.80 (0.55, 1.16)	**0.62 (0.47, 0.82)**	0.75 (0.50, 1.14)
Fair/poor	1.17 (0.65, 2.11)	1.11 (0.57, 2.17)	1.13 (0.68, 1.86)	1.40 (0.76, 2.58)	**0.52 (0.31, 0.86)**	0.49 (0.23, 1.05)
Education level (reference = not a college graduate)						
College graduate	0.92 (0.67, 1.26)	1.03 (0.72, 1.47)	**1.50 (1.14, 1.99)**	1.13 (0.79, 1.57)	**1.86 (1.45, 2.40)**	1.13 (0.77, 1.67)
Marital status (reference = married/partnered) (2)						
Divorced/widowed/single	1.21 (0.87, 1.69)	1.29 (0.87, 1.93)	**0.66 (0.47, 0.92)**	1.08 (0.67. 1.54)	**0.62 (0.47, 0.84)**	1.31 (0.82, 2.09)
Insurance (reference = private insurance)						
Medicaid	1.55 (0.81, 2.96)	1.28 (0.60, 2.73)	0.81 (0.42, 1.56)	0.93 (0.41, 2.09)	**0.35 (0.18, 0.67)**	**0.38 (0.16, 0.91)**
Medicare	0.82 (0.58, 1.15)	1.42 (0.67, 3.02)	**0.24 (0.16, 0.36)**	1.95 (0.74, 5.16)	**0.11 (0.07, 0.17)**	0.44 (0.17, 1.15)
Not insured	1.41 (0.48, 4.12)	0.98 (0.32, 2.99)	0.79 (0.27, 2.30)	0.68 (0.22, 2.16)	0.55 (0.21, 1.43)	0.53 (0.15, 1.84)
Income (reference < $50,000)						
> $50,000	0.80 (0.49, 1.31)	0.91 (0.59, 1.40)	**1.64 (1.19, 2.26)**	1.14 (0.74, 1.78)	**2.44 (1.82, 3.28)**	1.49 (0.90, 2.45)
Refused	0.81 (0.58, 1.13)	0.87 (0.50, 1.51)	0.93 (0.57, 1.52)	0.83 (0.46, 1.48)	1.33 (0.86, 2.05)	1.30 (0.67, 2.51)
Health literacy score	**0.91 (0.84, 0.98)**	**0.90 (0.83, 0.98)**	0.95 (0.88, 1.01)	**0.88 (0.81, 0.96)**	1.04 (0.98, 1.11)	1.07 (0.96, 1.18)
Surgery received (reference = lumpectomy)						
Mastectomy	Not included in model	Not included in model	Not included in model	Not included in model	**10.54 (7.15, 15.54)**	**16.3 (10.3, 25.9)**
Bilateral mastectomy	Not included in model	Not included in model	Not included in model	Not included in model	**34.01 (23.11, 50.21)**	**29.1 (18.5, 45.7)**

*Note:* In addition to the above listed variables, site of recruitment/cancer treatment was significant in both models. Significance indicated in bold.

### 3.3 Treatment Quality Care Coordination and Decision‐Making

Table 4 gives study participant responses to survey items regarding treatment quality and perceived care coordination during breast cancer treatment (unadjusted associations). We observed no differences in responses to items regarding treatment facilities (getting a second opinion; number of medical centers seen for treatment). We did see a difference in responses to items regarding care coordination. A higher proportion of women in higher BMI categories were more likely to report that their medical team always worked well together (e.g., 65% among BMI 19–24 kg/m^2^ vs. 73% among BMI 36^+^ kg/m^2^), as well as to report that their doctors seemed to be aware of treatments that other doctors had ordered (e.g., 56% among BMI 19–24 kg/m^2^ vs. 69% among BMI 36^+^ kg/m^2^). Because of these findings, we also examined whether the provider coordination scale varied by the BMI category and found no differences (*p* = 0.411; data not shown). When examining items related to care quality, we observed a difference in only one measure: women with obesity were less likely to report that the quality of their post‐diagnosis healthcare was excellent or very good than those without obesity (e.g., 85% among BMI 19–24 kg/m^2^ vs. 77% among BMI 36^+^ kg/m^2^). There were no differences in responses regarding provider communications, timely treatment, and access to specialists for treatment.

**TABLE 4:** Responses to survey items regarding treatment quality, provider coordination, and care coordination during breast cancer treatment, Share Thoughts on Breast Cancer Study, 2013–2014, *n* = 1198.

Covariate	Level	BMI 19–24	BMI 25–29	BMI 30–35	BMI 36+	*p* ^a^
		*N* = 417 (4)	*N* = 353 (3)	*N* = 262 (2)	*N* = 166 (1)	
Treating facililities						
Before beginning treatments, did you get a second opinion about treatment options for your breast cancer?	No	273 (66.3)	244 (70.1)	191 (73.2)	117 (70.9)	0.10
	Yes	139 (33.7)	104 (29.9)	70 (26.8)	48 (29.1)	
How many medical centers did you go to for breast cancer treatments? Breast cancer treatments include surgery to remove breast cancer, chemotherapy, radiation therapy, or hormone therapy	1 medical center	235 (57.3)	187 (53.7)	142 (54.8)	96 (57.8)	0.99
	2 medical centers	135 (32.9)	122 (35.1)	90 (34.7)	55 (33.1)	
	3 or more medical centers	40 (9.8)	39 (11.2)	27 (10.4)	15 (9.0)	
Coordination and responsiveness of care						
During your cancer treatment, was there one health professional who COORDINATED your cancer care?	No	131 (31.8)	87 (24.9)	54 (20.8)	31 (18.8)	0.079
	Yes	236 (57.3)	219 (62.8)	173 (66.8)	113 (68.5)	
	Do not know	45 (10.9)	43 (12.3)	32 (12.4)	21 (12.7)	
How often did the doctors, nurses, and other medical staff providing your care seem to work well together as a team?	Sometimes/never	30 (7.3)	10 (2.9)	12 (4.6)	8 (4.9)	**0.024**
	Usually	115 (27.9)	90 (25.6)	61 (23.5)	37 (22.6)	
	Always	267 (64.8)	251 (71.5)	187 (71.9)	119 (72.6)	
How often did your doctors seem to be aware of treatments for your breast cancer that other doctors had ordered?	Sometimes/never	35 (8.7)	20 (5.8)	17 (6.7)	7 (4.4)	**0.01**
	Usually	144 (35.8)	112 (32.4)	88 (34.9)	44 (27.3)	
	Always	223 (55.5)	214 (61.8)	147 (58.3)	110 (68.3)	
How often did you think that your health problems related to your breast cancer or its treatments were handled quickly enough?	Sometimes/never	30 (7.9)	31 (8.8)	19 (7.4)	13 (7.3)	0.71
	Usually	125 (30.4)	103 (29.3)	69 (26.7)	48 (29.3)	
	Always	256 (62.3)	217 (61.8)	170 (65.9)	103 (62.8)	
How often were you able to see the specialists such as cancer doctors you wanted to see for your breast cancer?	Sometimes/never	8 (1.9)	8 (2.3)	7 (2.7)	7 (4.3)	0.20
	Usually	78 (18.9)	67 (19.0)	51 (19.7)	33 (20.1)	
	Always	326 (79.1)	277 (78.7)	201 (77.6)	124 (75.6)	
How often did you know who to ask when you had any questions related to your breast cancer or its treatments?	Sometimes/never	35 (8.5)	24 (6.9)	21 (8.1)	13 (7.9)	0.53
	Usually	145 (35.0)	110 (31.4)	94 (36.2)	49 (29.9)	
	Always	234 (56.5)	216 (61.7)	145 (55.8)	102 (62.2)	
How often did you feel that your doctors, nurses, and other medical staff did everything they could to treat your health problems related to your breast cancer or its treatments?	Sometimes/never	17 (4.1)	12 (3.4)	9 (3.5)	8 (4.9)	0.39
	Usually	93 (22.6)	69 (19.8)	48 (18.5)	31 (18.9)	
	Always	302 (73.3)	268 (76.8)	203 (78.1)	125 (76.2)	
Overall care quality						
Overall, how would you rate the quality of your health care since you found out you had breast cancer?	Excellent/VG	353 (85.3)	288 (82.5)	209 (80.4)	127 (76.5)	**0.009**
	Good	47 (11.4)	48 (13.8)	45 (17.3)	26 (15.7)	
	Fair/poor	14 (3.4)	13 (3.7)	6 (2.3)	13 (7.8)	
Provider communication						
How often did your doctors listen carefully to you?	Sometimes/never	32 (7.8)	23 (6.6)	18 (6.9)	13 (8.0)	0.46
	Usually	107 (26.0)	85 (24.2)	75 (28.8)	48 (29.4)	
	Always	273 (66.2)	243 (69.2)	167 (64.2)	102 (62.6)	
How often did your doctors explain things in a way you could understand?	Sometimes/never	10 (2.4)	14 (4.0)	17 (6.6)	9 (5.5)	0.78
	Usually	125 (30.2)	100 (28.5)	74 (28.6)	35 (21.3)	
	Always	279 (67.4)	237 (67.5)	168 (64.9)	120 (73.2)	

^a^ Parametric *p*‐values calculated using the Mantel–Haenszel chi‐square test for trend. Bold values indicate statistically significance.

Finally, we investigated differences in decision‐making and decisional regret around breast cancer surgery. We observed several differences in the factors that were reported to be important in breast cancer treatment decision‐making by BMI (Table S2). In univariate analyses, a smaller proportion of women with in higher BMI categories reported that the following were very important when making surgical decisions: feeling bad about their bodies (e.g., 53% among BMI 19–24 kg/m^2^ vs. 46% among BMI 36^+^ kg/m^2^), interfering with their sex life in the long term (e.g., 42% among BMI 19–24 kg/m^2^ vs. 23% among BMI 36^+^ kg/m^2^), and allowing them to feel feminine (e.g., 50% among BMI 19–24 kg/m^2^ vs. 41% among BMI 36^+^ kg/m^2^). There were no significant differences in the extent to which concerns related to cancer recurrence differed by the BMI category. We also assessed differences between preferred and actual decision‐making during cancer treatment (i.e., was their actual role in decision‐making more active, the same as or more passive than their preferred role) and found no differences by the BMI category (*p* = 0.657; data not shown). Finally, when examining differences in overall decision‐making, decisional satisfaction and regret by BMI (Table S2), we observed no differences in overall satisfaction with treatment decisions, having enough support and information to make choices, and being clear about the best treatment choices. We did observe that a larger proportion of women with higher BMIs reported that treatment decisions were easy for them to make than those without and that they felt sure about what treatments to choose.

## 4 Discussion

In this study, we examined whether differences in GPC academic medical centers' breast cancer patients' treatments, related experiences, and decision‐making differed by BMI. Encouragingly, we observed that in this study, population of breast cancer patients treated by facilities within the GPC and patients' experiences of breast cancer treatment were largely similar across categories of BMI. While we did observe differences in surgical treatments received in univariate models, we were not able to detect statistically significant associations with BMI after adjustment for factors known to impact treatment received, including treatment location, patient age, health literacy, and health status (despite HR substantially lower than 1.0). Further, while we observed few differences in specific aspects of patient‐reported quality of care, including provider communication and access to specialist care, women with obesity were less likely to be overall satisfied with their care.

This study contributes to a growing body of literature exploring treatment differences as a potential explanation for observed survival disparities among breast cancer patients with obesity. While there is extensive literature describing complications and considerations for breast cancer treatment among those with obesity [14], there is considerably less evidence to indicate whether and how receipt of cancer treatment varies by weight or BMI. In a population‐based study in Geneva, Switzerland, women with obesity were more likely to present with advanced stage cancers and less likely to receive mastectomy than those without obesity after adjustment for age, health insurance type, and stage [22]. Conversely, in a single‐institution study in the USA, there was no difference in treatments received in multivariable‐adjusted models [6]. There is also some evidence to indicate that women with obesity may receive intentionally reduced doses of chemotherapy [11], despite a lack of evidence that this necessary [23]. In our study, we observed no difference in treatments received by BMI after adjustment for several important covariates, including, age, health status, insurance, and treatment/recruitment site. Unfortunately, we were unable to assess whether there were differences in what treatments participants were offered, reasons for treatment decision‐making, or whether these differed by BMI, as these questions were not included in the Share Thoughts on Breast Cancer survey. The small number of studies and mixed findings indicate the need for continued research on this topic.

There are several medical reasons why treatments received might vary by body size: obesity may increase the risk of surgical complications for mastectomy [24] in both implant‐based and autologous (“flap”) reconstruction [14], which could influence provider and patient decision‐making. Individuals with obesity may also experience different side effects of treatments, including endocrine and chemotherapies [25]. Further, patients with obesity may be more likely to have concurrent comorbidities that could affect their treatment, such as diabetes and cardiovascular disease [5]. Unfortunately, there is a startling lack of information specific to comorbidity prevalence among individuals with both obesity and breast cancer, although one single‐site study did indicate that patients with breast cancer and obesity were more likely than those without obesity to have a history of smoking, hypertension, and diabetes [6], and among our sample, participants with obesity were more likely to report a history of several conditions, including cardiovascular diseases, diabetes, and hypertension. If patients are managing comorbidities concurrent to their cancer diagnosis, it might explain our findings that women with obesity were more likely to report care coordination—in particular if comorbidities require coordination with non‐cancer care providers. Finally, a small number of qualitative studies indicate that patients with obesity may have stigmatizing experiences during their cancer care [26], which may impact desire to engage with healthcare professionals and/or maintain compliance with treatments.

The perception of stigmatizing care, which we were not able to assess in our study due to the use of previously collected survey data, may explain why we observed no difference by BMI in reports of specific quality metrics but lower satisfaction with care quality overall among patients with obesity. It is difficult to assess the impact of weight‐based stigma on cancer treatment decision‐making, treatments received, or potential impact on cancer survival disparities, in part because no studies quantitatively assess provider weight bias or patient experiences of weight stigma during cancer treatment [26]. Weight stigma is known to affect healthcare for people with obesity: a majority of individuals with obesity report experiencing weight‐based stigma from medical providers [27, 28, 29], and this impacts engagement with quality and outcomes of care [16, 28, 30]. Further, weight‐based stigma (implicit or explicit) may influence what treatment options are even presented to patients, with different (or lesser) treatment options offered to those with obesity. This may explain our univariate finding that women with obesity were more likely to receive lumpectomy, as well as findings around higher reported ease of decision‐making (i.e., treatment decisions may be easier for women with obesity if they are presented less options by their provider). Research is sorely needed to understand whether anti‐fat attitudes observed among other provider types [31] exist among cancer treatment providers, and whether these affect cancer treatment decision‐making or patient experiences of weight stigma in the cancer treatment setting.

Finally, we observed non BMI‐related findings that may be of interest. In particular, we observed an inverse association of health literacy with receipt of both mastectomy and CPM in multinomial models; i.e., those with higher health literacy were less likely to receive mastectomy or CPM. A small number of studies have suggested that lower health literacy is associated with a higher perception of breast cancer risk [32, 33], and since motivation for more extensive surgery is often a desire to decrease risk and worry about risk [34], it may be that women with higher health literacy are better able to accurately assess/understand their personal risk of local recurrence/contralateral breast cancer when counseled and so more likely to accept lumpectomy. Although higher education is associated with increased likelihood of receipt of CPM [35, 36, 37, 38], associations between treatment and health literacy have mostly been null [39], including another study in a GPC cohort [20].

This study has several limitations that warrant consideration in interpretation of the findings. First, there is a lack of racial and ethnic diversity in the Share Thoughts on Breast Cancer study sample; further, all patients in this sample received treatment at academic medical centers. Therefore, our findings may not be generalizable to those of diverse backgrounds and/or those who received treatment in community‐based settings. Importantly, the study may have been affected by survivor bias (i.e., individuals who did not survive long enough to be invited to participate in this study may be systematically different from those who did) and volunteer bias (i.e., those who self‐selected into the study may have different characteristics than the general population of breast cancer patients). Further, while we recognize that BMI as a measure of obesity is highly problematic [40, 41] and is not an adequate measure of individual health, this screening tool has demonstrated some utility in population‐based and epidemiologic studies, and it remains a commonly used clinical tool that may be used by clinicians in the process of making decisions regarding an individual's cancer treatment. For these reasons, we elected to use this variable as our primary exposure measure in analyses. As with all self‐reported data, there is potential for difficulties with recall to have resulted in misclassification; we do not expect such misclassification to have been differential by BMI. Given the constraints of the previously completed survey, we were also unable to directly assess experiences of weight stigma during treatment among our survey respondents; this may be important given evidence to suggest patient experiences of weight stigma during breast cancer treatment [26] and may have contributed to the lower overall perception of treatment quality among participants with obesity.

## 5 Conclusions

This study represents one of the few to examine whether and how breast cancer treatments received vary by obesity status, operationalized in this study using BMI. We found that there were no differences by BMI in treatments received after multivariable adjustment. Additionally, while there were few differences by BMI reported in terms of specific aspects of care quality, patients with obesity reported an overall lower quality of breast cancer care. Further research is needed to determine whether breast cancer treatment varies by body size and obesity in larger, more diverse samples and to determine reasons why treatments and treatment quality may vary including comorbidities, concerns regarding medical complication, patient preferences, and the potential role of provider attitudes toward individuals with obesity.

## Author Contributions

**Sarah H. Nash:** conceptualization (equal), formal analysis (equal), methodology (equal), supervision (equal), writing – original draft (equal), writing – review and editing (equal). **Elizabeth Verhage:** formal analysis (equal), investigation (equal), methodology (equal). **Bradley D. McDowell:** conceptualization (equal), formal analysis (equal), methodology (equal), supervision (equal), writing – review and editing (equal). **Joan Neuner:** methodology (equal), project administration (equal), writing – review and editing (equal). **Elizabeth Chrischilles:** methodology (equal), project administration (equal), writing – review and editing (equal). **Ingrid M. Lizarraga:** methodology (equal), writing – review and editing (equal). **Mary Schroeder:** conceptualization (equal), methodology (equal), writing – review and editing (equal).

## Consent

All participants provided informed consent for participation.

## Conflicts of Interest

The authors declare no conflicts of interest.

## Supporting information


Appendix S1.

## Data Availability

Data are available from the Share Thoughts on Breast Cancer study upon reasonable request to the University of Iowa College of Public Health Effectiveness Research Center.
