# Supplementary material for: Body Weight and Breast Cancer Treatment Experiences: Results From the Share Thoughts on a Breast Cancer Study
Source: Cancer Med. 2025 Feb 4;14(3):e70628. doi: 10.1002/cam4.70628 (PMC11794236; doi:10.1002/cam4.70628)
Supplement: Supplementary file 1 — Appendix S1. [file CAM4-14-e70628-s001.docx]

**Supplementary Table 1:** Results of multivariable analyses examining associations with surgical reconstruction among those who received mastectomy or bilateral mastectomy only, Share Thoughts on Breast Cancer study participants, Greater Plains Collaborative, 2013-2014, n = 494.

| **Model outcome →** | **Unadjusted OR** | **Multivariable**  **aOR** |
| --- | --- | --- |
| **Variable/Level ↓** |  |  |
| BMI *(reference = 19-24kg/m^2^) (4)* |  |  |
| 25-29 (3) | **0.56 (0.36, 0.88)** | 0.46 (0.21, 1.04) |
| 30-35 (2) | **0.48 (0.29, 0.81)** | 0.75 (0.38, 1.45) |
| 36+ (1) | **0.35 (0.19, 0.65)** | 0.74 (0.42, 1.31) |
| Age category *(reference = <45 years) (4)* |  |  |
| 45-54 (3) | 0.55 (0.27, 1.11) | 0.59 (0.28, 1.26) |
| 55-64 (2) | **0.17 (0.09, 0.34)** | **0.22 (0.11, 0.48)** |
| 65+ (1) | **0.03 (0.01, 0.07)** | **0.08 (0.02, 0.28)** |
| Self-reported health *(reference = Excellent/very good) (3)* |  |  |
| Good (2) | **0.45 (0.30, 0.67)** | 0.64 (0.38, 1.08) |
| Fair/poor (1) | **0.38 (0.20, 0.72)** | 0.62 (0.25, 1.53) |
| Education level *(reference = not a college graduate) (2)* |  |  |
| College graduate (1) | **0.87 (1.96, 4.20)** | 1.40 (0.84, 2.33) |
| Marital status *(reference = married/partnered) (2)* |  |  |
| Divorced/widowed/single (1) | **0.45 (0.30, 0.68)** | 1.10 (0.60, 2.00) |
| Insurance *(reference = private insurance) not recoded* |  |  |
| Medicaid (2) | **0.16 (0.07, 0.36)** | **0.36 (0.13, 0.98)** |
| Medicare (3) | **0.08 (0.05, 0.14)** | 0.57 (0.18, 1.85) |
| Not insured (1) | 0.31 (0.09, 1.09) | 0.47 (0.11, 2.02) |
| Income *(reference <$50,000) (3)* |  |  |
| > $50,000 (2) | **4.16 (2.73, 6.32)** | 1.66 (0.89, 3.10) |
| Refused (1) | **2.19 (1.18, 4.08)** | 1.77 (0.77, 4.03) |
| Health literacy score | **1.18 (1.08, 1.29)** | 1.12 (0.99, 1.27) |
| Surgery received *(reference = mastectomy) (2)* |  |  |
| Bilateral mastectomy (1) | **3.23 (2.21, 4.72)** | 1.60 (0.98, 2.59) |

*in addition to the above listed variables, site of recruitment/cancer treatment was significant in both models. **significance indicated in bold

| **Supplementary Table 2.** Decision making around breast cancer surgical treatment, Share Thoughts on Breast Cancer Study, 2013-2014, n = 1198. | | | | | | |
| --- | --- | --- | --- | --- | --- | --- |
| **Covariate** | **Level** | **BMI 19-24 N=417** | **BMI 25-29 N=353** | **BMI 30-35 N=262** | **BMI 36+ N=166** | **P-value*** |
| **When decisions were made about breast cancer surgery, how important was it that the type of surgery you had….** |  |  |  |  |  |  |
| Would keep you from worrying about the cancer coming back? | Not important | 4 (1.0) | 4 (1.1) | 3 (1.2) | 2 (1.2) | 0.06 |
|  | Somewhat | 18 (4.4) | 16 (4.6) | 19 (7.3) | 14 (8.6) |  |
|  | Very important | 386 (94.6) | 328 (94.3) | 237 (91.5) | 147 (90.2) |  |
| Would reduce the chance of cancer coming back? | Not important | 3 (0.7) | 3 (0.9) | 6 (2.3) | 2 (1.2) | 0.15 |
|  | Somewhat | 7 (1.7) | 6 (1.7) | 6 (2.3) | 4 (2.5) |  |
|  | Very important | 395 (97.5) | 339 (97.4) | 246 (95.3) | 157 (96.3) |  |
| Would allow you to avoid the possibility of a second surgery to remove the cancer? | Not important | 9 (2.2) | 12 (3.5) | 7 (2.7) | 4 (2.5) | 0.52 |
|  | Somewhat | 34 (8.4) | 23 (6.6) | 22 (8.5) | 17 (10.4) |  |
|  | Very important | 362 (89.4) | 312 (89.9) | 229 (88.8) | 142 (87.1) |  |
| Would not make you feel bad about your body, like it was disfigured? | Not important | 88 (21.8) | 83 (23.9) | 78 (30) | 48 (30.0) | **0.008** |
|  | Somewhat | 104 (25.7) | 98 (28.2) | 68 (26.2) | 39 (24.4) |  |
|  | Very important | 212 (52.5) | 167 (48.0) | 114 (43.8) | 73 (45.6) |  |
| Would not interfere with your sex life in the long term? | Not important | 124 (31.2) | 136 (40.0) | 111 (43.5) | 78 (48.8) | **<0.001** |
|  | Somewhat | 106 (26.6) | 82 (24.1) | 52 (20.4) | 46 (28.7) |  |
|  | Very important | 168 (42.2) | 122 (35.9) | 92 (36.1) | 36 (22.5) |  |
| Would allow you to feel feminine? | Not important | 99 (24.4) | 107 (31.2) | 87 (33.9) | 49 (30.4) | **0.021** |
|  | Somewhat | 104 (25.7) | 88 (25.7) | 56 (21.8) | 46 (28.6) |  |
|  | Very important | 202 (49.9) | 148 (43.1) | 114 (44.4) | 66 (41.0) |  |
| Would allow you to avoid exposing yourself to radiation? | Not important | 131 (33.1) | 129 (38.1) | 101 (40.4) | 65 (41.4) | 0.08 |
|  | Somewhat | 89 (22.5) | 70 (20.6) | 56 (22.4) | 24 (15.3) |  |
|  | Very important | 176 (44.4) | 140 (41.3) | 93 (37.2) | 68 (43.3) |  |
| Would allow you to avoid going back and forth to radiation treatments? | Not important | 159 (40.4) | 147 (44.3) | 104 (41.8) | 64 (40.5) | 0.84 |
|  | Somewhat | 72 (18.3) | 51 (15.4) | 54 (21.7) | 26 (16.5) |  |
|  | Very important | 163 (41.4) | 134 (40.4) | 91 (36.5) | 68 (43.0) |  |

| **Supplementary Table 2.** Decision making and decisional satisfaction and regret regarding breast cancer treatment, Share Thoughts on Breast Cancer Study, 2013-2014, n = 1198. | | | | | | |
| --- | --- | --- | --- | --- | --- | --- |
| **Covariate** | **Level** | **BMI 19-24 N=417** | **BMI 25-29 N=353** | **BMI 30-35 N=262** | **BMI 36+ N=166** | **P-value*** |
| **Now, thinking about all the treatment decisions you made for your breast cancer, please mark how much each statement is true for you.** |  |  |  |  |  |  |
| I was clear about the best choice for me | Strongly disagree/disagree (1) | 17 (4.2) | 12 (3.4) | 11 (4.2) | 8 (4.8) | 0.99 |
|  | Neither agree nor disagree (2) | 21 (5.1) | 18 (5.2) | 8 (3.1) | 8 (4.8) |  |
|  | Strongly agree/agree (3) | 371 (90.7) | 319 (91.4) | 240 (92.7) | 150 (90.4) |  |
| The decision was easy for me to make | Strongly disagree/disagree | 83 (20.3) | 52 (14.9) | 28 (10.9) | 18 (10.9) | **<0.001** |
|  | Neither agree nor disagree | 48 (11.8) | 29 (8.3) | 23 (8.9) | 17 (10.3) |  |
|  | Strongly agree/agree | 277 (67.9) | 267 (76.7) | 206 (80.2) | 130 (78.8) |  |
| I felt sure about what to choose | Strongly disagree/disagree | 43 (10.6) | 29 (8.3) | 19 (7.4) | 19 (11.4) | **0.033** |
|  | Neither agree nor disagree | 64 (15.8) | 30 (8.6) | 15 (5.8) | 13 (7.8) |  |
|  | Strongly agree/agree | 299 (73.6) | 289 (83.0) | 223 (86.8) | 134 (80.7) |  |
| I felt sure that I had enough support and advice to make a choice | Strongly disagree/disagree | 12 (2.9) | 12 (3.4) | 7 (2.7) | 8 (4.8) | 0.86 |
|  | Neither agree nor disagree | 25 (6.1) | 15 (4.3) | 13 (5.1) | 7 (4.2) |  |
|  | Strongly agree/agree | 370 (90.9) | 321 (92.24 | 237 (92.2) | 151 (90.1) |  |
| I feel that I made an informed choice | Strongly disagree/disagree | 8 (2.0) | 10 (2.9) | 4 (1.6) | 4 (2.4) | 0.71 |
|  | Neither agree nor disagree | 19 (4.7) | 15 (4.3) | 8 (3.1) | 7 (4.2) |  |
|  | Strongly agree/agree | 379 (93.3) | 321 (92.8) | 244 (95.3) | 155 (93.4) |  |
| I am satistfied with my decisions | Strongly disagree/disagree | 12 (2.9) | 10 (2.9) | 8 (3.1) | 4 (2.4) | 0.25 |
|  | Neither agree nor disagree | 28 (6.8) | 17 (4.9) | 5 (1.9) | 9 (5.4) |  |
|  | Strongly agree/agree | 369 (90.2) | 321 (92.2) | 244 (94.9) | 153 (92.2) |  |
| I made decisions before I was ready | Strongly disagree/disagree | 310 (76.7) | 266 (77.1) | 195 (76.2) | 121 (73.8) | 0.18 |
|  | Neither agree nor disagree | 60 (14.9) | 38 (11.0) | 32 (12.5) | 20 (12.2) |  |
|  | Strongly agree/agree | 34 (8.4) | 41 (11.9) | 29 (11.3) | 23 (14.0) |  |

*Parametric p-values calculated using Mantel-Haenszel chi-square test for trend.
